# Nodule-specific *AhPUGN1.1* positively regulates nodulation in peanuts

**DOI:** 10.1007/s42994-025-00222-7

**Published:** 2025-07-02

**Authors:** Haitong He, Weiqing Liu, Yiwei Xu, Xuerui Fang, Wei Zhang, Zhaosheng Kong, Lixiang Wang

**Affiliations:** Shanxi Hou Ji Laboratory, College of Agriculture, Shanxi Agricultural University, Taigu, 030801 China

**Keywords:** Peanut, Rhizobia, Symbiotic nitrogen fixation, *AhPUGN1.1*

## Abstract

Peanut (*Arachis hypogaea*) is a widely cultivated legume crop that can fix nitrogen by forming root nodules with compatible rhizobia. The initiation and formation of these nodules require complex molecular communication between legumes and rhizobia, involving the precise regulation of multiple legume genes. However, the mechanism underlying nodulation in peanuts remains poorly understood. In this study, we identified a gene associated with nodulation in peanuts, named *Peanut unique gene for nodulation 1.1* (*AhPUGN1.1*). Multiple lines of evidence indicate that *AhPUGN1.1* is primarily expressed in peanut nodules. Silencing or knocking out *AhPUGN1.1* in peanut resulted in fewer nodules, as well as lower fresh weight and nitrogenase activity, while overexpressing *AhPUGN1.1* significantly enhanced nodulation ability and nitrogenase activity. Modulating the expression of *AhPUGN1.1* also influenced the expression levels of genes associated with the Nod factor signaling pathway and infection via crack entry. Comparative transcriptome analysis revealed that *AhPUGN1.1* likely regulates peanut nodulation by affecting the expression of genes involved in the cytokinin and calcium signaling pathways. Our data thus show that *AhPUGN1.1* acts as a crucial regulator promoting symbiotic nodulation in peanuts.

**Supplementary Information:**

The online version contains supplementary material available at 10.1007/s42994-025-00222-7.

## Introduction

Peanut (*Arachis hypogaea*) is a traditional oilseed crop cultivated in tropical, subtropical, and temperate regions. Its seeds are rich in protein, vegetable oil, and phytochemicals for human consumption. Leguminous plants like peanut typically derive 60–65% of their nitrogen quota from symbiotic fixation of atmospheric dinitrogen (N_2_) (Lavin et al. 2005). This symbiotic relationship with specific rhizobia can substantially diminish the need for nitrogen fertilization, which is essential for sustainable agricultural development and ecological preservation (Sprent 2007).

Nodulation is a complex biological program involving direct interactions between signals derived from rhizobia and legumes (Karmakar et al. 2018). In peanuts, the base of the lateral root, with its dense cluster of unicellular root hairs, is the best place to study how peanut root primordia and nodules form (Bhattacharjee et al. 2022; Bonaldi et al. 2011). The "crack-entry" mode of rhizobial infection primarily occurs at the loosely structured cell walls at the base of multicellular root hairs. Rhizobial cells diffuse into the intercellular space, move to the root cortex by altering cell walls, initiate nodule formation in the lateral root cortex, and multiply in infested cells, which divide repeatedly to form nodules (Karmakar et al. 2018). Once the nodule matures, the host cells stop dividing and form a bacteroid that converts atmospheric N_2_ to ammonia via catalysis by nitrogen-fixing enzymes, providing a source of nitrogen to the plant (Raul et al. 2022).

Symbiotic nodulation in peanuts involves multiple signaling pathways from initial infection to nodule maturation (Sprent and James 2007). A study on the slow-growing mutant *Bradyrhizobium* sp. SEMIA 6144 V2 *nodC*, lacking the nodulation factor (Nod), revealed the essential role of Nod in the crack-entry mode of infection of peanut (Ibañez and Fabra 2011). The host extracellular polysaccharide receptor EPS receptor 3 (EPR3), along with other host proteins such as Nod factor receptor 5 (NFR5), Nodulation signaling pathway 1 (NSP1), and Nodule inception (NIN), has been reported to help establish the symbiotic relationship between peanuts and rhizobia (Ferguson et al. 2018; Shu et al. 2020). The phosphorylated Symbiosis Receptor-like Kinase (SYMRK) mediates rhizobial infection in peanut through a well-defined phosphorylation cascade. Upon perception of rhizobial Nod factors during early symbiotic signaling, SYMRK undergoes rapid phosphorylation, initiating a downstream signaling network that coordinates nodule organogenesis (Saha et al. 2016). Furthermore, the calcium (Ca^2+^)/calmodulin-dependent protein kinase CCaMK forms a core molecular hub connecting signal recognition and nodule development by phosphorylating the transcriptional regulator CYCLOPS (Singh et al. 2014). When Nod is recognized by receptors of the lysine motif (LysM) family, plant roots trigger perinuclear Ca^2+^ oscillations to activate CCaMK, leading to the phosphorylation of specific serine residues such as Ser-50 and Ser-154 in CYCLOPS (Das et al. 2019). Phosphorylated CYCLOPS binds to promoter regions of downstream target genes, including the palindromic sequence of the CYCLOPS response element (CYC-RE), to regulate the expression of key symbiotic genes (Akamatsu et al. 2022). Among the products of these genes, the GRAS-family transcription factor NIN initiates nodule primordium formation and infection thread development, while Early nodulin 40 (ENOD40) promotes cortical cell division to facilitate rhizobial invasion (Battaglia et al. 2014). Additionally, the histidine kinase HK1 integrates symbiotic signals with the cytokinin signaling pathway to enhance symbiosis and suppress plant defense responses, establishing an immunoprotective niche for rhizobia (Sharma et al. 2020). This phosphorylation cascade converts Ca^2+^ signals into a gene expression program through precise spatiotemporal regulation, ultimately determining the efficiency of nodule formation and nitrogen fixation (Cerri et al. 2017; Oldroyd 2013; Singh et al. 2014). The phytohormone cytokinin plays a role in nodule primordium formation by modulating the expression of the peanut cytokinin receptor Arabidopsis histidine kinase 1 (AHK1) (Kundu and DasGupta 2018). Additionally, ethylene-related genes such as *Ethylene response factor* (*ERF*) and the ethylene signaling component *Ethylene-insensitive 2* (*EIN2*) are highly expressed in peanut nodules, helping regulate nodule progenitor cell division, differentiation, and nodule development (Sharma et al. 2020; Vernié et al. 2008).

To better understand symbiotic nodulation in peanuts, we sought to identify additional nodule-specific genes and uncover their potential roles during nodulation. Here we report on a unique gene, *Peanut unique gene for nodulation 1.1* (*AhPUGN1.1*), which is specifically expressed in peanut nodules. Overexpression of *AhPUGN1.1* led to the formation of more and heavier nodules with greater nitrogenase activity, whereas its knockdown and knockout had the opposite effects. We show that AhPUGN1.1 is localized in the nucleus and can activate the expression of key nodulation genes, suggesting it may be a transcription factor or transcriptional activator that regulates nodulation at the very early stages in peanuts. These findings establish *AhPUGN1.1* as a crucial gene for symbiotic nodulation in peanuts, paving the way for improved nitrogen fixation efficiency.

## Results

### *AhPUGN1.1* is differentially expressed during peanut nodulation

To explore the genes related to nitrogen fixation during nodulation in peanuts, we performed a differential gene expression analysis using a publicly available transcriptome deep sequencing (RNA-seq) dataset (Raul et al. 2022). We identified several genes as being mainly expressed in nodules, revealing their potential roles during peanut nodulation. Of these, we focused in this study on a gene we named *AhPUGN1.1*; this gene is present only in the peanut genome, with no ortholog detected in those of other legumes, suggesting it may be a species-specific gene (Fig. S1A–D). A whole-genome scan detected three homologs (*AhPUGN1.2*: XM_025766943.2, *AhPUGN1.3*: XM_025766944.2, and *AhPUGN1.4*: XM_025830201.2) distributed on chromosomes 10 and 20 in the tetraploid genome of peanuts (Figs. S2, S3).

An analysis of the *AhPUGN1.1* promoter region (2-kb region upstream of the 5' untranslated region) revealed the presence of a nodulation-specific *cis*-regulatory element, NODCON2GM, present in four copies (Fig. S3B). A previous report indicates that NODCON2GM is a conserved motif in the promoters of nodulin-related genes (Stougaard et al. 1990). Mutation or deletion of NODCON2GM leads to lower expression of these genes (Stougaard et al. 1990; Jørgensen et al. 1991). The presence of several copies of the NODCON2GM motif in the promoter region of *AhPUGN1.1* and its homologs suggests that these genes are likely involved in nodulation.

**Fig. 1:**
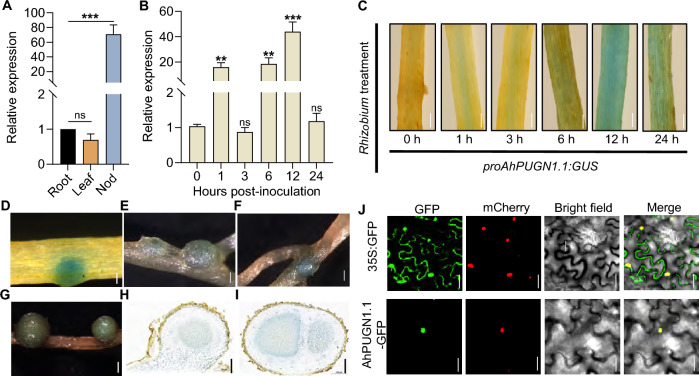
Expression of *AhPUGN1.1* and subcellular localization of AhPUGN1.1. **A** Relative expression of *AhPUGN1.1* in different tissues, as determined by RT-qPCR. **B** Relative expression of *AhPUGN1.1* in peanut roots at 0 h, 1 h, 3 h, 6 h, 12 h, or 24 h after inoculation with the rhizobium CCBAU.051107. **C, D** GUS staining of hairy roots (**C**) or nodule primordia (**D**) from transgenic peanut plants carrying the *proAhPUGN1.1:GUS* reporter construct. **E, F** GUS staining of young root nodule. **G** Representative photograph of mature peanut nodules. Bars, 1 mm. **H, I** Cross sections of nodules from transgenic peanut roots harboring the *proAhPUGN1.1:GUS* construct at 10 (**H**) or 24 (**I**) days post-inoculation (dpi). Bars, 200 μm. **J** Subcellular localization of AhPUGN1.1-GFP in *N. benthamiana* leaves. Bars, 70 μm. All data are means ± standard deviation (SD) from three independent experiments. Significant differences were determined by Student’s *t-*test (*P < 0.05; **P < 0.01; ***P < 0.001; ns, not significant)

To investigate the expression pattern of *AhPUGN1.1* during rhizobial infection and nodule development, we examined its transcript levels in leaves, roots, and nodules after inoculating roots with *Bradyrhizobium arachidis* strain CCBAU.051107. We observed that AhPUGN1.1 is predominantly expressed in roots with nodules. We also investigated the spatial expression pattern of *AhPUGN1.1* in peanuts via a reporter construct consisting of the *AhPUGN1.1* promoter region driving expression of the *β-glucuronidase* (*GUS*) reporter gene (*proAhPUGN1.1:GUS*). A histochemical staining assay detected GUS staining in root nodules, confirming *AhPUGN1.1* promoter activity in these tissues (Fig. 1A and Fig. S4A and B). Moreover, *AhPUGN1.1* expression was rapidly induced at 1 h post-inoculation (hpi) with *B. arachidis* CCBAU.051107, peaking at 12 hpi, whereas its homologs (*AhPUGN1.2*, *AhPUGN1.3*, and *AhPUGN1.4*) did not show significant but variable expression changes in response to infection (Fig. 1B and Fig. S4C), suggesting a role for *AhPUGN1.1* during early nodulation events.

GUS histochemical staining in hairy roots transformed with the *proAhPUGN1.1:GUS* construct confirmed the temporal expression pattern of *AhPUGN1.1* observed by RT-qPCR (Fig. 1B, C). Indeed, we detected GUS activity in nodule primordia (Fig. 1D), bumps (Fig. 1E, F), and mature nodules (Fig. 1G). Paraffin sections of stained nodules revealed GUS signals in the infected zones and nitrogen-fixing zones of nodules at 10 days post-inoculation (dpi) and 24 dpi (Fig. 1H, I). Turning to the protein encoded by *AhPUGN1.1*, the online tool NLSExplorer predicted the presence of a nuclear localization signal (NLS) with the sequence IIWQYWTIKNWGRKIKDWYYSNPN (Fig. S5). To assess its subcellular localization, we generated the *AhPUGN1.1-GFP* construct, encoding a fusion of AhPUGN1.1 with the green fluorescent protein (GFP) driven by the cauliflower mosaic virus (CaMV) 35S promoter, and infiltrated this construct into the leaves of *N. benthamiana* plants via *Agrobacterium tumefaciens*-mediated infiltration. Confocal microscopy observations detected green fluorescence signals from AhPUGN1.1-GFP in the nucleus, confirming the nuclear localization of AhPUGN1.1 (Fig. 1J). These results suggest that *AhPUGN1.1* might be a nodule-specific gene regulating nodule formation in rhizobium-infected peanut.

### *AhPUGN1.1* promotes nodulation in peanuts

**Fig. 2:**
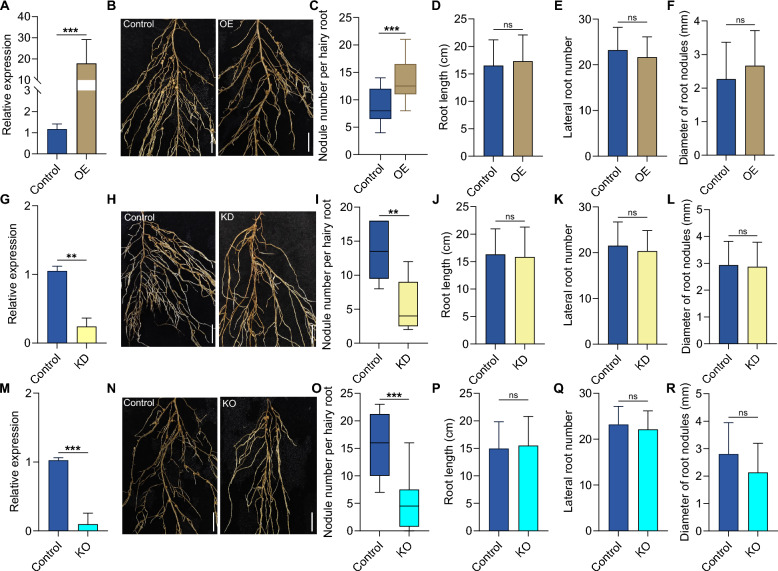
*AhPUGN1.1* positively regulates peanut nodulation. **A** Relative expression of *AhPUGN1.1* in control and *AhPUGN1.1*-OE transgenic hairy roots (*n* = 10). **B** Representative photographs of peanut nodules from control and *AhPUGN1.1*-OE hairy roots. Bars, 5 mm. **C** Number of nodules on control and *AhPUGN1.1*-OE roots (*n* = 10). Root length (**D**), lateral root number (**E**), and diameter of root nodule (**F**), for the control group and *AhPUGN1.1*-OE group (*n* = 10). **G** Relative expression of *AhPUGN1.1* in control and *AhPUGN1.1*-KD transgenic hairy roots (*n* = 10). **H** Representative photographs of control (empty vector) and *AhPUGN1.1*-KD roots. Bars, 5 mm. **I** Number of nodules on empty vector and *AhPUGN1.1*-KD roots (*n* = 10). Root length (**J**), lateral root number (**K**), and diameter of root nodules (**L**), for the control group and the *AhPUGN1.1-*KD group (*n* = 10). **M** Relative expression of *AhPUGN1.1* in control and *AhPUGN1.1*-KO transgenic hairy roots (*n* = 10). **N** Representative photographs of peanut roots from control and *AhPUGN1.1*-KO hairy roots. Bars, 5 mm. **O** Number of nodules on empty vector and *AhPUGN1.1*-KO hairy roots (*n* = 10). Root length (**P**), lateral root number (**Q**), and diameter of root nodules (**R**), for the control and KO groups (*n* = 10). All data are means ± SD from three independent biological replicates. Significant differences were determined by Student’s *t-*test (*P < 0.05; **P < 0.01; ***P < 0.001; ns, not significant)

To investigate the role of *AhPUGN1.1* in nodulation, we generated *AhPUGN1.1* overexpression (OE) and RNA interference (KD) plants using *Agrobacterium rhizogenes*-mediated hairy root transformation. The expression of *AhPUGN1.1* was significantly higher in *AhPUGN1.1*-OE hairy roots compared with control hairy roots transformed with the empty construct (Fig. 2A). These *AhPUGN1.1*-OE transgenic roots produced more nodules than the control roots at 28 dpi (Fig. 2B, C). In the *AhPUGN1.1*-KD transgenic roots, *AhPUGN1.1* transcript levels were significantly lower than those in control roots, while the expression levels of its homologous genes were not clearly affected (Fig. 2G and Fig. S6). The number of nodules visible on *AhPUGN1.1*-KD transgenic roots was much lower than that on control roots, suggesting that lower *AhPUGN1.1* transcript levels compromise nodule production in peanuts (Fig. 2H, I). In addition, we utilized clustered regularly interspaced short palindromic repeats (CRISPR)/CRISPR-associated nuclease 9 (Cas9)-mediated gene editing to knockout *AhPUGN1.1* in hairy roots and obtained three types of mutation for *AhPUGN1.1*, either a 1-bp deletion or two distinct 1-bp insertions at the target site (Fig. S7). The *AhPUGN1.1*-KO hairy roots also exhibited lower *AhPUGN1.1* expression levels and fewer nodules (Fig. 2M–O). These results indicate that *AhPUGN1.1* may positively regulate nodulation by promoting nodule generation, at least when tested in the hairy root system. To examine whether *AhPUGN1.1* influences root system architecture, we measured root length, lateral root number, and the diameter of nodules in control, *AhPUGN1.1*-OE, *AhPUGN1.1*-KD, and *AhPUGN1.1*-KO hairy roots at 28 dpi. However, we detected no statistically significant differences (Fig. 2D–F, J–L, P–R).

**Fig. 3:**
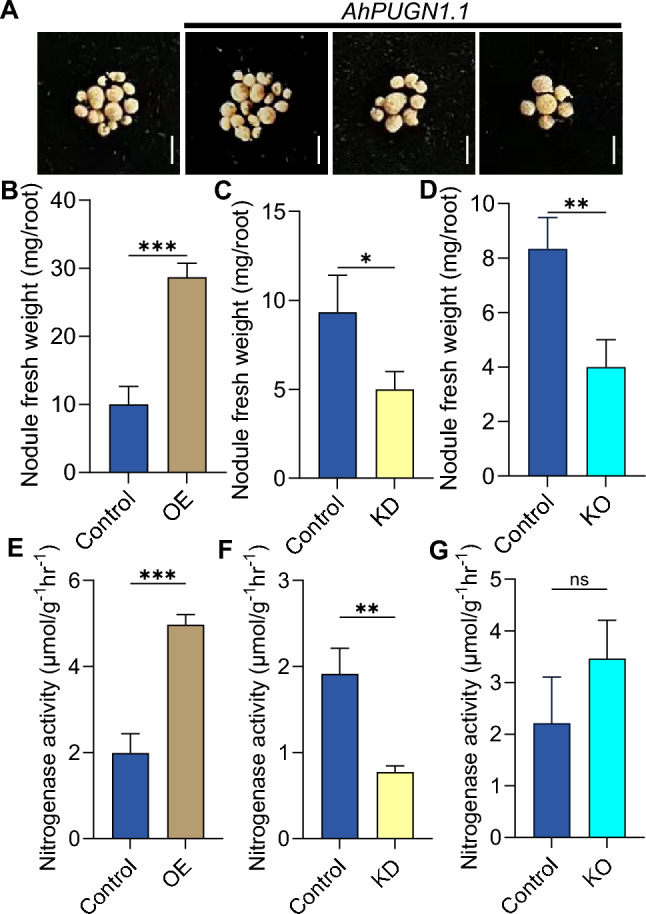
Characterization of the nodules from *AhPUGN1.1*-OE, *AhPUGN1.1*-KD, and *AhPUGN1.1*-KO hairy roots. **A** Representative photographs of the nodules produced on control, *AhPUGN1.1*-OE, *AhPUGN1.1*-KD, and *AhPUGN1.1*-KO hairy roots. Bars, 1 cm. Fresh weight of nodules from control and *AhPUGN1.1*-OE (**B**), *AhPUGN1.1*-KD (**C**), and *AhPUGN1.1*-KO (**D**) hairy roots. Nitrogenase activity from the nodules of control and *AhPUGN1.1*-OE (**E**), *AhPUGN1.1*-KD (**F**), and *AhPUGN1.1*-KO (**G**) hairy roots (*n* = 30). Data are means ± SD from three biological replicates (*n* = 10). Significant differences were determined by Student’s *t-* test (*P < 0.05; **P < 0.01; ***P < 0.001; ns, not significant)

We detached fresh nodules from the control, *AhPUGN1.1*-OE, *AhPUGN1.1*-KD, and *AhPUGN1.1*-KO hairy roots to investigate their fresh weight and measure their nitrogenase activity (Fig. 3). Compared with control roots, the fresh weight of nodules from *AhPUGN1.1-OE* roots was significantly higher, while that from *AhPUGN1.1*-KD and *AhPUGN1.1*-KO roots was significantly lower (Fig. 3A–D). We then used these fresh nodules to measure nitrogenase activity via acetylene reduction assays. The nodules detached from *AhPUGN1.1*-OE hairy roots showed significantly higher nitrogenase activity compared with nodules from control roots, while the nodules detached from *AhPUGN1.1*-KD roots had a lower nitrogenase activity. However, the nitrogenase activity of nodules was not significantly different between *AhPUGN1.1*-KO and control roots (Fig. 3E–G). These results further demonstrate the positive regulatory role of *AhPUGN1.1* in nodule formation.

### *AhPUGN1.1* regulates nodule formation through modulating the expression of Nod factor signaling genes

**Fig. 4:**
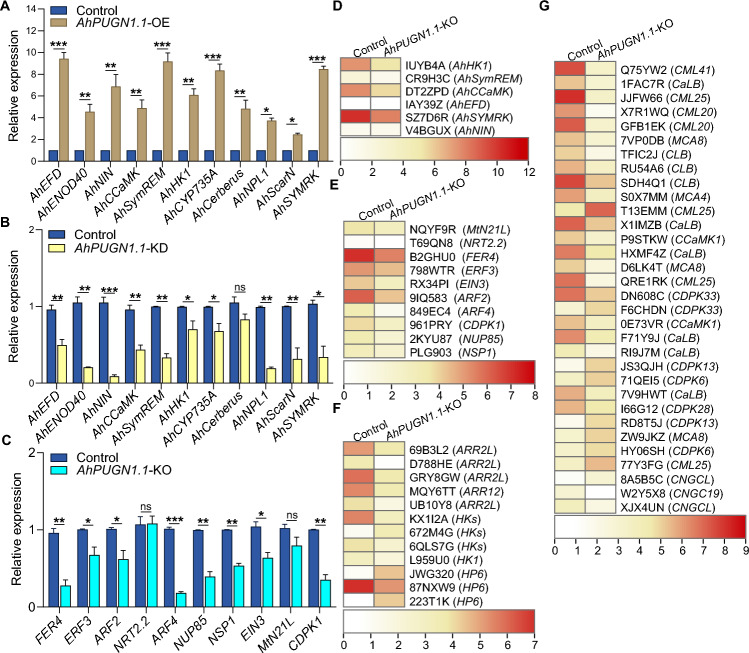
Nodulation-related marker genes and differentially expressed genes that may be regulated by *AhPUGN1.1*. **A** RT-qPCR analysis of nodule-associated marker gene expression in control and *AhPUGN1.1*-OE hairy roots at 28 dpi. **B** Quantification of nodule-associated gene transcripts by RT-qPCR in *AhPUGN1.1*-KD roots at 28 dpi.** C** Expression levels of nodulation-related genes in control and *AhPUGN1.1*-KO roots, as determined by RT-qPCR. **D** Heatmap representation of expression levels for nodulation marker genes in the control and *AhPUGN1.1*-KO hairy roots. Heatmap representation of expression for nodulation-related (**E**), cytokinin signaling (**F**), and calcium signaling (**G**) genes in control and *AhPUGN1.1*-KO hairy roots. All data are means ± SD from three independent experiments. Significant differences were determined by two-tailed Student’s *t-* test (*P < 0.05; **P < 0.01; ***P < 0.001)

Nod factor (NF) signaling results in widespread transcriptional reprogramming that leads to coordinated infection and nodule organogenesis (Roy et al. 2020). Th us, we checked whether *AhPUGN1.1* might promote nodulation by regulating the expression of NF signaling genes. To this end, we examined the transcript levels of genes involved in the NF pathway, such as *AhCCaMK*, *AhHK1*, *AhNIN*, *Symbiotic remorin* (*AhSymREM*), *AhEFD*, and *AhENOD40* (Karmakar et al. 2018; Kundu and DasGupta 2018; Das et al. 2019). RT-qPCR showed significantly higher transcript levels of these genes in *AhPUGN1.1*-OE roots and lower levels in *AhPUGN1.1*-KD roots versus controls (Fig. 4A, B).

Since the genes *Cytochrome P450 735A* (*CYP735A*), *Cerberus*, *NPL1*, *SCAR-Nodulation* (*ScarN*), and *SYMRK* are reported to be involved in intercellular infection processes (Bhattacharjee et al. 2022; Fabre et al. 2015; Montiel et al. 2021; Qiu et al. 2015; Sharma et al. 2020; Xie et al. 2012), we analyzed the expression levels of their peanut orthologs in control, *AhPUGN1.1*-OE, and *AhPUGN1.1*-KD transgenic hairy roots. The transcript levels of *AhCYP735A*, *AhNPL1*, *AhScarN*, and *AhSYMRK* were significantly higher in the *AhPUGN1.1*-OE hairy roots and lower in the *AhPUGN1.1*-KD hairy roots than in the control roots (Fig. 4A, B). These genes play distinct roles in the crack-entry infection mode. *LjCYP735A* of *Lotus japonicus*, encoding a cytokinin hydroxylase, is crucial for cytokinin homeostasis during intercellular infection by the *Rhizobium* strain IRBG74, as its mutation leads to delayed nodulation and a greater number of uninfected white nodules (Lin et al. 2021; Reid et al. 2017). Cerberus, a U-box protein in *L. japonicus*, is involved in cytoskeletal rearrangements, with mutants showing defective infection threads during intracellular infection and significantly fewer nodulation events during intercellular infection (Yano et al. 2009). Nodule pectate lyase (NPL1) and the cytoskeleton component ScarN are essential for infection thread progression and bacterial invasion, as their mutants produce fewer nodules during intercellular infection (Xie et al. 2012; Qiu et al. 2015). The receptor LjSYMRK, a core component of the NF signaling pathway, is indispensable for both infection modes, as its mutants fail to form nodules (Madsen et al. 2003; Capoen et al. 2005).

We further performed RNA-seq on control and *AhPUGN1.1*-KO hairy roots. Among the NF pathway-related genes mentioned above, *AhSymREM*, *AhHK1*, and *AhCCaMK* expression was downregulated in the *AhPUGN1.1*-KO hairy roots, consistent with the observed changes in the *AhPUGN1.1*-KD roots (Fig. 4B, D). However, *ERF required for nodule differentiation* (*AhEFD*) and *AhNIN* expression showed no significant difference from the control roots (Fig. 4D).

### Comparative RNA-seq analysis reveals the positive regulation of peanut nodulation by AhPUGN1.1

To further explore the regulatory role of AhPUGN1.1 in peanut nodulation, we explored the transcriptome of control and *AhPUGN1.1*-KO roots. Among the differentially expressed genes (DEGs), 13,196 were upregulated (56.6%) and 10,111 were downregulated (43.4%) (Fig. S8A). Gene Ontology (GO) term analysis revealed distinct functional enrichment patterns between DEGs: upregulated genes showed a significant enrichment in the “negative regulation of mitotic cell cycle” pathway, while downregulated genes demonstrated notable associations with “cellular response to nitrogen levels,” “cellular response to nitrogen starvation,” and “cell surface receptor signaling pathway” (Fig. S8B and C). A Kyoto Encyclopedia of Genes and Genomes (KEGG) pathway analysis further indicated an enrichment of DEGs in the “alanine, aspartate, and glutamate metabolism” pathway (Fig. S8D). Among the DEGs, genes related to nodulation, such as B2GHU0 (*Ferritin 4* [*FER4*]), 798WTR (*ERF3*), 9IQ583 (*ARF2*), T69QN8 (*Nitrate transport 2.2* [*NRT2.2*]), 849EC4 (*Auxin-related factor 4* [*ARF4*]), 2KYU87 (*Nucleoporin 85* [*NUP85*]), PLG903 (*NSP1*), RX34PI (*EIN3*), NQYF9R (*MtN21-like* [*MtN21L*]), and 961PRY (*CDPK1*), were downregulated in *AhPUGN1.1*-KO roots (Fig. 4C, E). Furthermore, knocking out *AhPUGN1.1* in hairy roots also affected the expression of *Ca*^*2*+^*-dependent protein kinase* (*CDPK*), *Cyclic nucleotide gated channel* (*CNGC*), and *Response regulator* (*RR*) family members (Fig. 4F, G). These genes have roles in either the Ca^2+^ or cytokinin signaling pathways, which regulate the initiation and maturation of nodules in legumes (Lin et al. 2021; Liu et al. 2022).

## Discussion

### *AhPUGN1.1* regulates nodule development in peanuts

Symbiotic nitrogen fixation (SNF) is considered to be the most efficient system in the ecosystem, providing a major nitrogen source for the growth and development of legumes (Zhao et al. 2021). Despite considerable progress in understanding SNF and its underlying regulatory mechanisms, much remains to be discovered regarding the details involved in peanut nodulation. In this study, we identified and characterized the gene *AhPUGN1.1* related to peanut nodule formation. We showed that *AhPUGN1.1* is mainly expressed in peanut root nodules and positively regulates nodulation.

As SNF is energy intensive, nodule number is finely controlled by nodulation formation-related genes and the auto-regulated nodulation (AON) signaling pathway (Li et al. 2022). NIN is an important transcription factor controlling nodule organogenesis and nodule number (Soyano et al. 2014; Yoro et al. 2014). *ENOD40* is a non-coding RNA required for cellular dedifferentiation and cortical cell division during nodule progenitor base formation. AhEFD and SymREM are both responsible for bacterial release and differentiation (Charon et al. 1997; Crespi et al. 1994; Lefebvre et al. 2010; Tóth et al. 2012; Vernié et al. 2008). In this study, we determined that the expression levels of *AhNIN*, *AhSymREM*, *AhEFD*, and *AhENOD40* were lower in *AhPUGN1.1-*KD roots relative to control roots (Fig. 4B). At the same time, the *AhPUGN1.1-*KD roots harbored fewer nodules. Based on these results, we propose AhPUGN1.1 as a crucial regulator promoting nodule development, contributing to the efficient nitrogen-fixing capacity of peanut root nodules.

### *AhPUGN1.1* regulates peanut nodulation by affecting the calcium and cytokinin signaling pathways

Legumes engage in nitrogen-fixing symbiosis with rhizobia, with Ca^2+^ proposed to be a major secondary messenger. Oscillations in Ca^2+^ levels are generated during the early stages of rhizobial infection (Tang et al. 2020). The perception of the generated Ca^2+^ signals involves the coordinated action of Ca^2+^ receptors, specifically CCaMKs and CDPKs (Martín and Busconi 2000). The oscillating Ca^2+^ signals are transduced and amplified through successive phosphorylation events, culminating in their specific recognition by CCaMK (Liu et al. 2022). This recognition triggers the phosphorylation of the transcriptional regulator AhCYCLOPS, which regulates the expression of downstream genes such as *AhHK1*, *AhNIN*, and *AhENOD40* (Kouchi et al. 2010; Tirichine et al. 2007). Consequently, this regulatory pathway influences nodule formation, nitrogen fixation, growth, development, immunity, and stress responses in peanut (Sinharoy and DasGupta 2009). We established that the expression of two *CCaMK* genes was lower in *AhPUGN1.1*-KO transgenic hairy roots than in control roots (Fig. 4G), suggesting that *AhPUGN1.1* may modulate peanut nodulation by adjusting Ca^2+^ signaling.

Nodule initiation is largely dependent on the local production of the phytohormone cytokinin (Boivin et al. 2016; Heckmann et al. 2011; Mathesius et al. 2000). Rhizobia can produce cytokinins as well, allowing root cortical cells to successfully dedifferentiate and proliferate during the initial stages of nodulation (Chen et al. 2022; Sasaki et al. 2014). Plants perceive cytokinins via HKs and transmit the signal to RRs through authentic histidine phosphotransferases (AHPs), propagating the signal by activating the expression of primary cytokinin-responsive genes (Kieber and Schaller 2018). Infection by rhizobia can induce the expression of cytokinin sensor genes, such as *MtCYTOKININ RESPONSE 1* (*MtCRE1*) in *Medicago truncatula* and *LOTUS HISTIDINE KINASE 1* (*LHK1*) in *L. japonicus* (Kieber and Schaller 2018; van Zeijl et al. 2015), which activate the cytokinin signaling pathway necessary for nodulation. Additional research has revealed that cytokinins may act downstream of CCaMK in the NF signaling pathway (Gonzalez-Rizzo et al. 2006; Murray et al. 2007; Tirichine et al. 2007). We noted the upregulation of *HK*s and *RR*s, and the downregulation of *HK*s and *AHP*s in the *AhPUGN1.1*-KO hairy roots (Fig. 4F). These results indicate that *AhPUGN1.1* potentially serves as an important regulator of cytokinin signaling, thereby modulating nodulation by adjusting Ca^2+^ signaling in peanuts.

In summary, we identified a previously uncharacterized gene in peanut, named *AhPUGN1.1*, and investigated its function and underlying mechanism during nodulation. Multiple lines of evidence revealed that AhPUGN1.1 positively affects nodulation in peanuts. Transcriptome analysis indicated that it may regulate signaling pathways related to calcium and cytokinins, underscoring its role in promoting symbiotic nodulation in peanuts. The identification and utilization of *AhPUGN1.1* may offer promising prospects for enhancing nitrogen fixation capacity in peanuts and perhaps other legume crops.

## Materials and methods

### Plant materials, growth conditions, and preparation of rhizobia

Seeds of the peanut variety “four-grain red” were germinated in sterilized vermiculite and cultured in growth chambers under a 16-h light/8-h dark photoperiod at 25 °C and with a relative humidity of 70%. When the first true leaves fully expanded, the peanut seedlings were inoculated with *Bradyrhizobium arachidis* CCBAU.051107 (GenBank: GCA_900116675.1) by applying a bacterial culture (OD_600_ = 0.08–0.1) around the roots. Approximately 35 mL bacterial solution was applied to each pot for inoculation (Chen et al. 2022).

### RNA extraction and gene expression analysis

The roots, leaves, and nodules of peanut seedlings were collected at 14 days after inoculation for RNA extraction. Total RNA was extracted according to the method of the TranZol kit (TransGen Biotech, China). First-strand cDNA was synthesized by reverse transcription according to the instructions of the *TransScript*^®^ One-Step gDNA Removal and cDNA Synthesis SuperMix kit. RT-qPCR was performed using the MonAmp™ SYBR^®^ Green qPCR Mix (MonAmp) according to the manufacturer's protocol on a Bio-Rad CFX96 instrument. Relative gene expression values were calculated using the 2^−ΔΔCt^ method and were normalized to the expression levels of the reference gene *AhActin*. Three biological replicates were analyzed. The primers used in this study are listed in Table S1 and Fig. S9.

### Plasmid construction

The full-length *AhPUGN1.1* coding sequence was cloned into the vector pSuper1300-GFP at the *Hin*dIII and *Sal*I sites (NEB, USA) to generate the pSuper1300-GFP-*AhPUGN1.1* construct. The full-length *AhPUGN1.1* coding sequence was amplified and inserted into the *Hind*III and *Sal*I restriction sites (NEB, USA) of the pCAMBIA1300-GFP vector using T4 DNA ligase to obtain the *AhPUGN1.1*-OE overexpression plasmid. The 1512-bp coding sequence fragment of *AhPUGN1.1* was inserted into the pK7GWIW-GFP vector using the Thermo Fisher Gateway™ LR Clonase™ II Enzyme Mix to generate the *AhPUGN1.1*-KD silencing construct. The 2000-bp promoter fragment of *AhPUGN1.1* (up to the 5′ untranslated region) was cloned into the pMDC162 vector via LR recombination, generating the *proAhPUGN1.1:GUS* construct. For the construction of the CRISPR knockout construct, single guide RNAs (sgRNAs) were designed using the online tool Crispr-P (http://crispr.hzau.edu.cn/CRISPR2/). One sgRNA with a high score was chosen and its sequence cloned into the pKSE401-GFP vector using the vector pCBC-DT1T2 as a template to generate the *AhPUGN1.1*-KO construct.

### Subcellular localization

The pSuper1300-GFP-*AhPUGN1.1* plasmid was introduced into Agrobacterium (*Agrobacterium rhizogenes*) strain K599. An Agrobacterium culture for a positive colony containing the pSuper1300-GFP-*AhPUGN1.1* plasmid was mixed in equal volume with an Agrobacterium culture harboring the nuclear marker vector pHBT-mCherry-NOS. The mixture was centrifuged at 1000 g for 1 min at room temperature to collect the bacterial pellet, which was resuspended in 1 mL of 10 mM MgCl_2_ to which 1 μL of 100 µM acetosyringone was added. After standing with no shaking for 3 h, the mixed Agrobacterium cell suspension was infiltrated into the leaves of *Nicotiana benthamiana* plants that had been cultured under a 16-h light/8-h dark photoperiod. Forty-eight hours after infiltration, a laser confocal scanning microscope was used to detect and capture the fluorescence signals (Bonaldi et al. 2011).

### Peanut hairy root transformation

*Agrobacterium rhizogenes* strain K599 was used for peanut hairy root transformation as described by Cheng et al. (2021). Three days after seed germination, the peanut seedlings were inoculated with *A. rhizogenes* at a cut site below the hypocotyl. The inoculated seedlings were transferred into pots filled with vermiculite and grown in a growth chamber. Two weeks after transplanting, positive transformation events were identified by checking for GFP fluorescence in the roots using a LUYOR-3415RG dual-wavelength portable fluorescent protein observation lamp. Positive transgenic plants were transplanted to fresh pots and inoculated with rhizobia 2 days later. Nodulation status was assessed 28 days after inoculation (Yuan et al. 2016).

### Histochemical GUS staining

Roots inoculated with *A. rhizogenes* harboring the *proAhPUGN1.1:GUS* construct were collected and stained overnight at 37 °C using X-Gluc solution (1 mg/mL, Coolaber, China) for GUS activity detection. Subsequently, 75% (v/v) ethanol was used to destain the tissues. The roots and nodules were prepared as described by Ge et al. (2024). Images were captured using a digital camera mounted on an ECLIPSE Ci-S microscope (Maikeney Instruments Co., Ltd., Nanjing, China).

### Nitrogenase activity measurements

Nitrogenase activity was measured using the acetylene reduction assay (ARA) (Montes-Luz et al. 2023). Approximately 0.3 g of freshly collected peanut root nodules (28 days post-transformation) was placed in a sealed glass vial, and 10% of the headspace gas was replaced with high-purity acetylene (C_2_H_2_, 99.999%; product catalog no. 80002861, Sinopharm Chemical Reagent Co., Ltd.). The enzymatic reaction was allowed to take place at 28 °C for 60 min and then terminated by placing the vials at 4 °C for 10 min. The production of ethylene (C_2_H_4_), the reaction product, was quantified using a gas chromatograph equipped with a flame ionization detector (FID), with nitrogen as the carrier gas at a flow rate of 30 mL/min. The retention times for acetylene and ethylene were 2.3 min and 1.8 min, respectively.

For quantitative analysis, an ethylene standard curve was constructed using several concentrations (10, 20, 40, 60, 80, and 100 μL). The response factor (*K*, μL/peak area) was determined via linear regression, and nitrogenase activity was calculated accordingly. The experiment included three biological replicates, and the standard curve exhibited a correlation coefficient (*R*^2^) of > 0.99 to ensure data reliability.

### Transcriptome deep sequencing processing and analysis

Total RNA was extracted from the roots of control and *AhPUGN1.1*-KO plants at 10 days after inoculation with *B. arachidis* CCBAU.051107 using a column-based method. RNA purity (OD_260/280_ ≥ 1.8, OD_260/230_ ≥ 1.0) was verified on a Nanodrop 2000, integrity was assessed by agarose gel electrophoresis, and RNA integrity number (RIN) of the RNA samples was determined on an Agilent 2100 system. For each sample, at least 1 μg total RNA was used, with a starting concentration ≥ 35 ng/μL. Polyadenylated mRNA was enriched using Oligo(dT) magnetic beads, followed by first-strand cDNA synthesis with random hexamers and second-strand synthesis to generate sticky-ended double-stranded cDNA, which was then end repaired (blunt-ended) using End Repair Mix, A-tailed at the 3′-end for adapter ligation, size selected after adapter ligation, and PCR amplified to produce the final library. Bridge PCR cluster generation was performed on a cBot system, and sequencing was conducted on an Illumina Novaseq 6000 platform as paired-end 150-bp reads. During data analysis, raw reads were quality checked using FastQC (https://www.bioinformatics.babraham.ac.uk/projects/fastqc/). Reads were filtered using Trimmomatic and aligned to the *Arachis hypogaea* reference genome with HISAT2 (http://ccb.jhu.edu/software/hisat2/index.shtml) and TopHat2 (http://tophat.cbcb.umd.edu/) (total mapping rate > 65%). Gene expression levels were quantified by HTSeq-count and normalized via Fragments Per Kilobase of transcript per Million mapped fragments (FPKM). Differentially expressed genes (DEGs) were identified using DESeq2 (threshold: |log_2_FC|≥ 1, adjusted *p* value < 0.05). Gene ontology (GO) functional enrichment analysis was performed with GoTool (significance: false discovery rate [FDR] < 0.05); Kyoto encyclopedia of genes and genomes (KEGG) pathway analysis followed the same criteria (significance: uncorrected *p* value < 0.05).

## Supplementary Information

Below is the link to the electronic supplementary material.

Supplementary file1 (DOCX 20332 KB)

## Data Availability

The datasets generated and analyzed during this study are available from the corresponding author on reasonable request.
