# Supplementary material for: Nodule-specific AhPUGN1.1 positively regulates nodulation in peanuts
Source: aBIOTECH. 2025 Jul 2;6(3):542–53. doi: 10.1007/s42994-025-00222-7 (PMC12454708; doi:10.1007/s42994-025-00222-7)
Supplement: Supplementary file 1 — Supplementary file1 (DOCX 20332 KB) [file 42994_2025_222_MOESM1_ESM.docx]

**Nodule-specific *AhPUGN1.1* positively regulates nodulation in peanuts**

Haitong He^1^, Weiqing Liu^1^, Yiwei Xu^1^, Xuerui Fang^1^, Wei Zhang^1^, Zhaosheng Kong^1^*, Lixiang Wang^1^*

^1^Shanxi Hou Ji Laboratory, College of Agriculture, Shanxi Agricultural University, Taigu 030801, China

*Correspondence: Lixiang Wang, lxwang@sxau.edu.cn

Zhaosheng Kong, zskong@sxau.edu.cn

| **Primer names** | **Sequence** |
| --- | --- |
| qRT-*AhNIN*-Forward | GGTACCTATGAGGAGGAGATCAGCC |
| qRT-*AhNIN*-Reverse | AGCATTAGAGAGATGATCAAGTTGTGG |
| qRT-*AhHK1*-Forward | GAGGTCTTAAAGCCATTGTGGTTGATGG |
| qRT-*AhHK1*-Reverse | CAACTGCTTGATAGAGATTCTCTTC |
| qRT-*AhEFD*-Forward | CAATTCCCAATTGGTGCTGGAACAATTGC |
| qRT-*AhEFD*-Reverse | GACCCAATTGAGCAAAGTTCAATTGATCCAC |
| qRT-*AhPUGN1.1*-Forward | CCCAATGATAATGTGGAAGTTG |
| qRT-*AhPUGN1.1*-Reverse | ATGTGTACTAGTCTCTGGTGG |
| qRT-*AhPUGN1.4*-Forward | TGCAGATAATGTGGAAGTTG |
| qRT-*AhPUGN1.4*-Reverse | AGGATCATTACCCTGATGA |
| qRT-*AhPUGN1.3*-Forward | CCCAATGATAATGTGGAAG |
| qRT-*AhPUGN1.3*-Reverse | GTGTTGTTCTGGTTGTGG |
| qRT-*AhPUGN1.2*-Forward | CGTGAATTTCCCAATGCAGAT |
| qRT-*AhPUGN1.2*-Reverse | GGATTATGTGGATGGGTATTTGG |
| qRT-*AhCCamK*-Forward | GCCAAAGATGTTCAAATGGACCCT |
| qRT-*AhCCamK*-Reverse | CCTGAGACTTTCAATTTCCTCTTC |
| qRT-*AhENOD40*-Forward | GGCAAAAATCCATCCATGGTTC |
| qRT-*AhENOD40*-Reverse | GCCTTTTTGTGACTTGCCGG |
| qRT-*AhSymREM*-Forward | GACTAAAATAGACAACAGGGC |
| qRT-*AhSymREM*-Reverse | GCAGTAAAGCATGAAAGAATTTTC |
| qRT-*AhSYMRK*-Forward | CAGATCAGCTTCTGCAAGTGAAGG |
| qRT-*AhSYMRK*-Reverse | GAAATGTGCCCCTTATCAGATACACTTTG |

**Table S1 Primers used in this study**

**
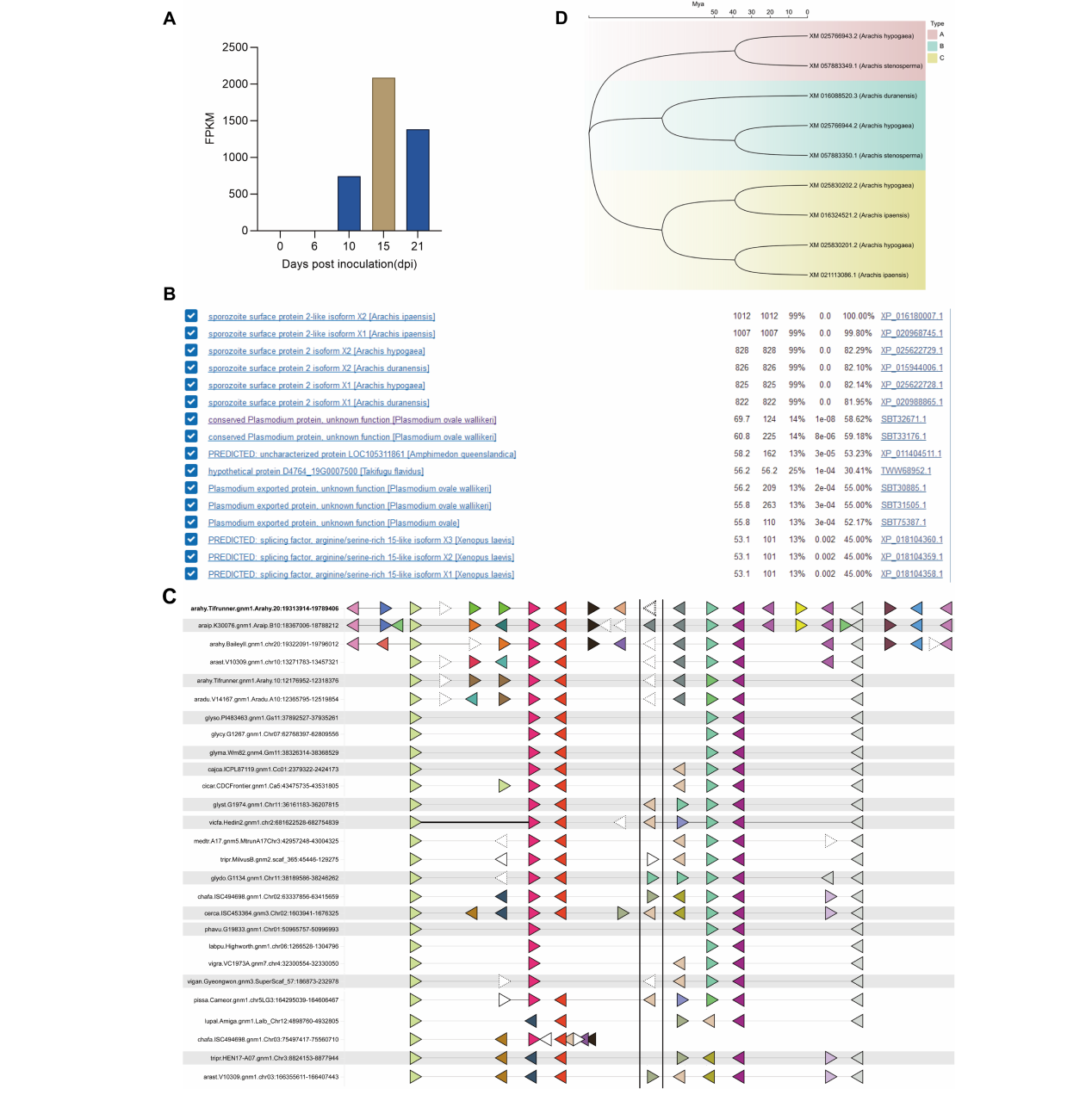
**

****Fig. S1 *AhPUGN1.1* is predominantly expressed in peanut nodules and may be specifically present in peanuts.** **A** study by Raul et al. (2022) examined the expression of *AhPUGN1.1* during peanut nodule development. The dataset (MPMI-05-21-0122-R_ST2) includes complete transcriptome data that has been submitted to NCBI (accession ID: PRJNA596350). **B** BLAST analysis was conducted across all species in NCBI using the AhPUGN1.1 protein sequence. **C** Micro-synteny analysis of AhPUGN1.1 was performed within legume species. **D** Phylogenetic analysis was carried out to investigate AhPUGN1.1 homologs in other peanut species.**


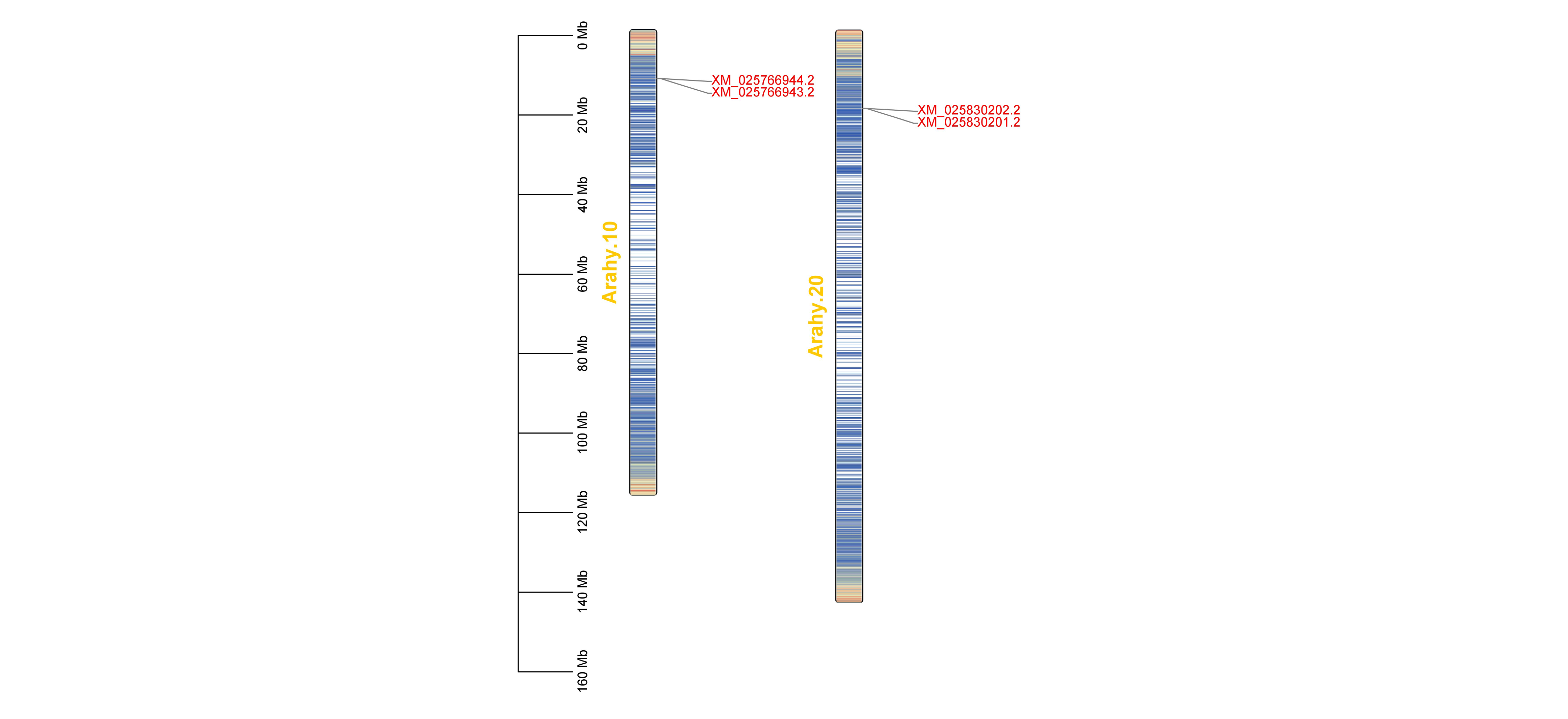


****Fig. S2** Chromosomal distribution of *AhPUGN1.1* and its homologous genes.**

**
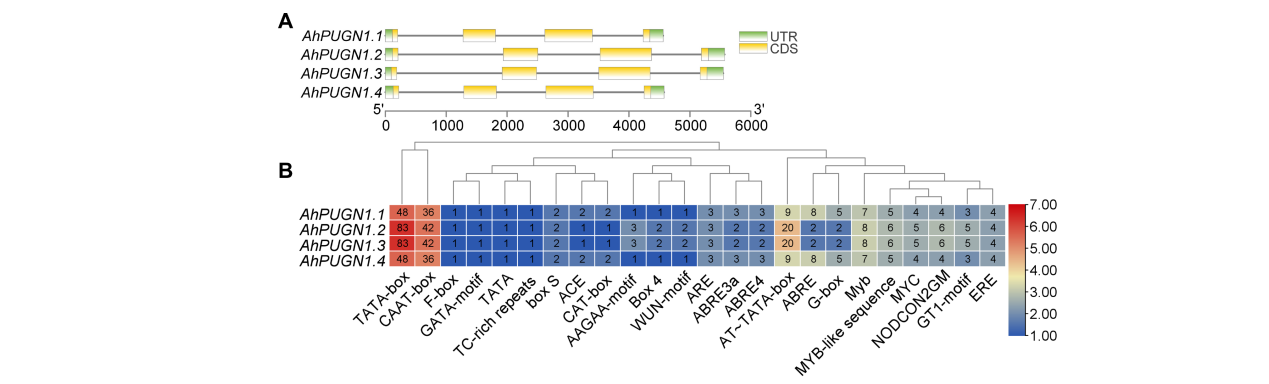
**

****Fig. S3 Bioinformatics analysis of *AhPUGN1.1* and its homologs.** A** Gene structure **of *AhPUGN1.1* and its homologs.** **B** *cis*-elements distribution in the promoter region of ***AhPUGN1.1***.


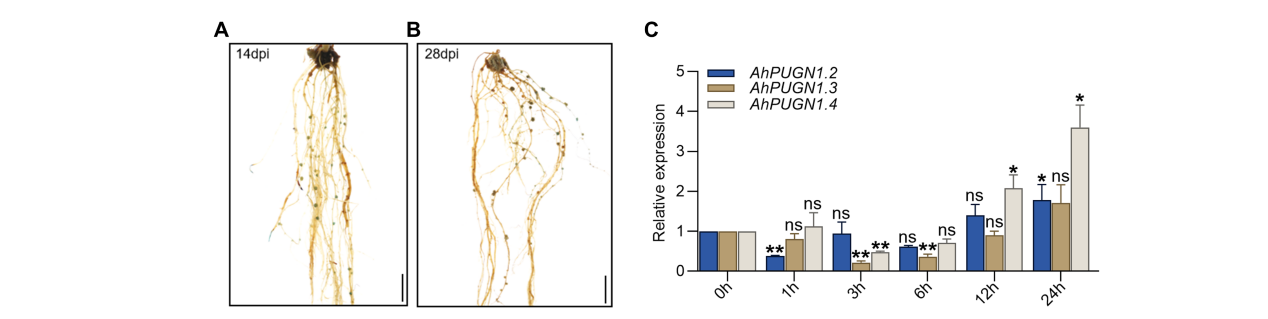


****Fig. S4** Expression analysis of *AhPUGN1* genes in response to rhizobium inoculation.** GUS activity in *pro*AhPUGN1.1*::GUS* expressed roots at 14 DPI (**A**), and 28 DPI (**B**). Bar = 5 mm. **C** Expression pattern of AhPUGN1.2, AhPUGN1.3, and AhPUGN1.4 at different time points after rhizobium inoculation. Normalized with *AhActin*, data represent mean ± SD of 3 biological replicates (*P < 0.05, **P < 0.01, ns indicates statistically non-significant, two-tailed Student's *t*-test).

**
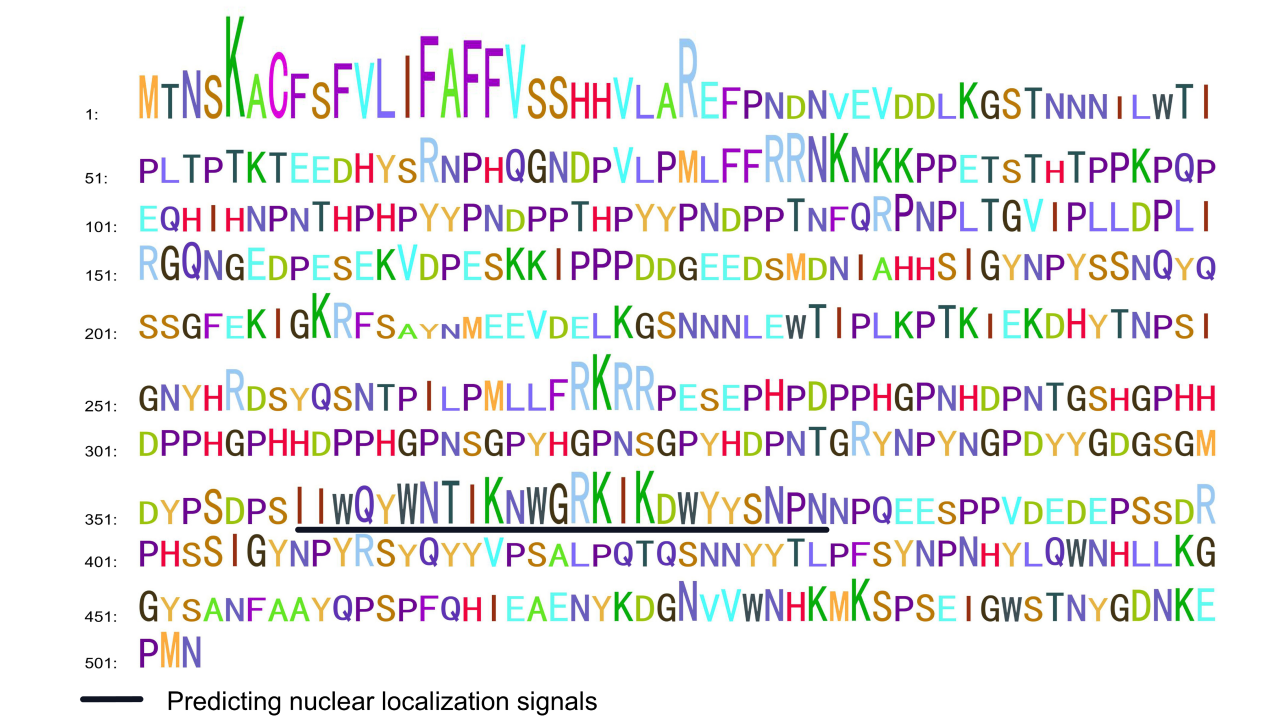
**

****Fig. S5** AhPUGN1.1 prediction of nuclear localization sequence.** NLSExplorer (http://www.csbio.sjtu.edu.cn/bioinf/NLSExplorer/index.html).

**
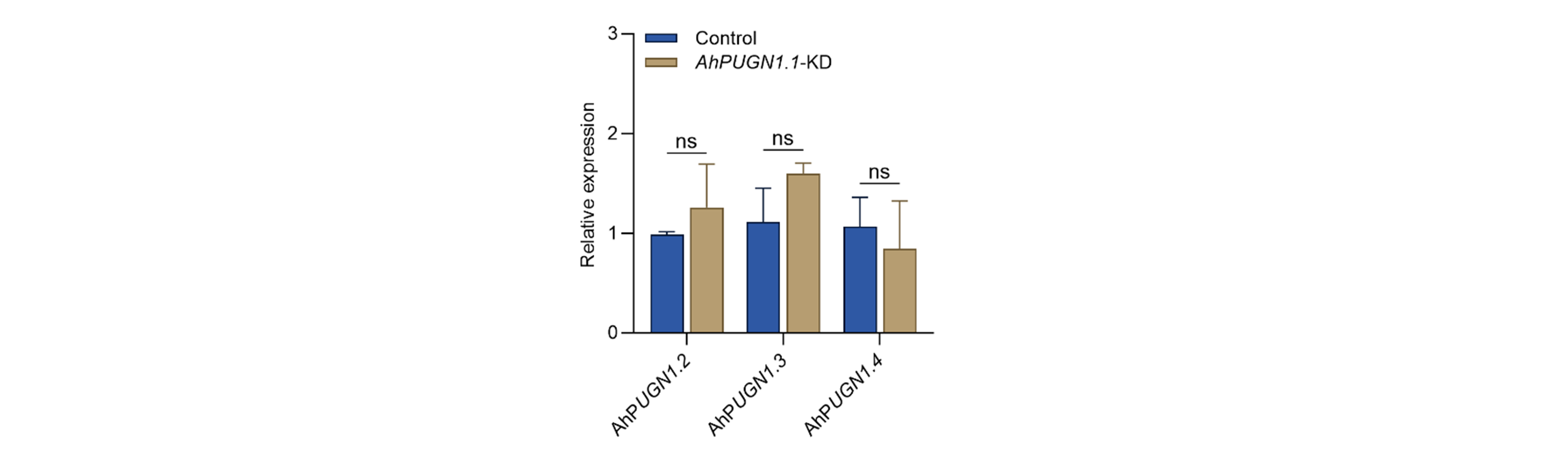
**

****Fig. S6 Relative expression levels of *AhPUGN1.2*, *AhPUGN1.3*, and *AhPUGN1.4* in *AhPUGN1.1*-KO roots.** “ns” indicates no significant difference compared to control (*P* > 0.05). Data represent means ± SD (*n* = 3, two-tailed Student's *t*-test).**


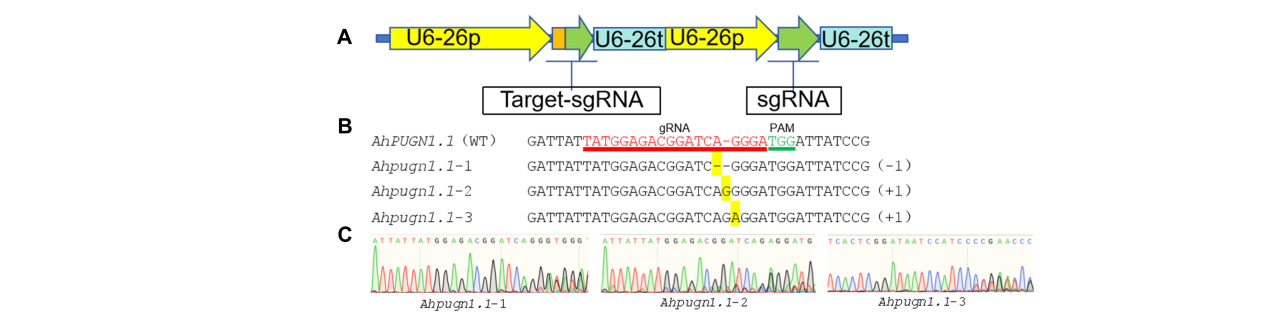


**Fig. S7 CRISPR-Cas9 based editing of *AhPUGN1.1.* A** Vector construction schematic diagram. **B** Mutations identified on *AhPUGN1.1* target. **C** Sequencing chromatogram of mutated *AhPUGN1.1*.


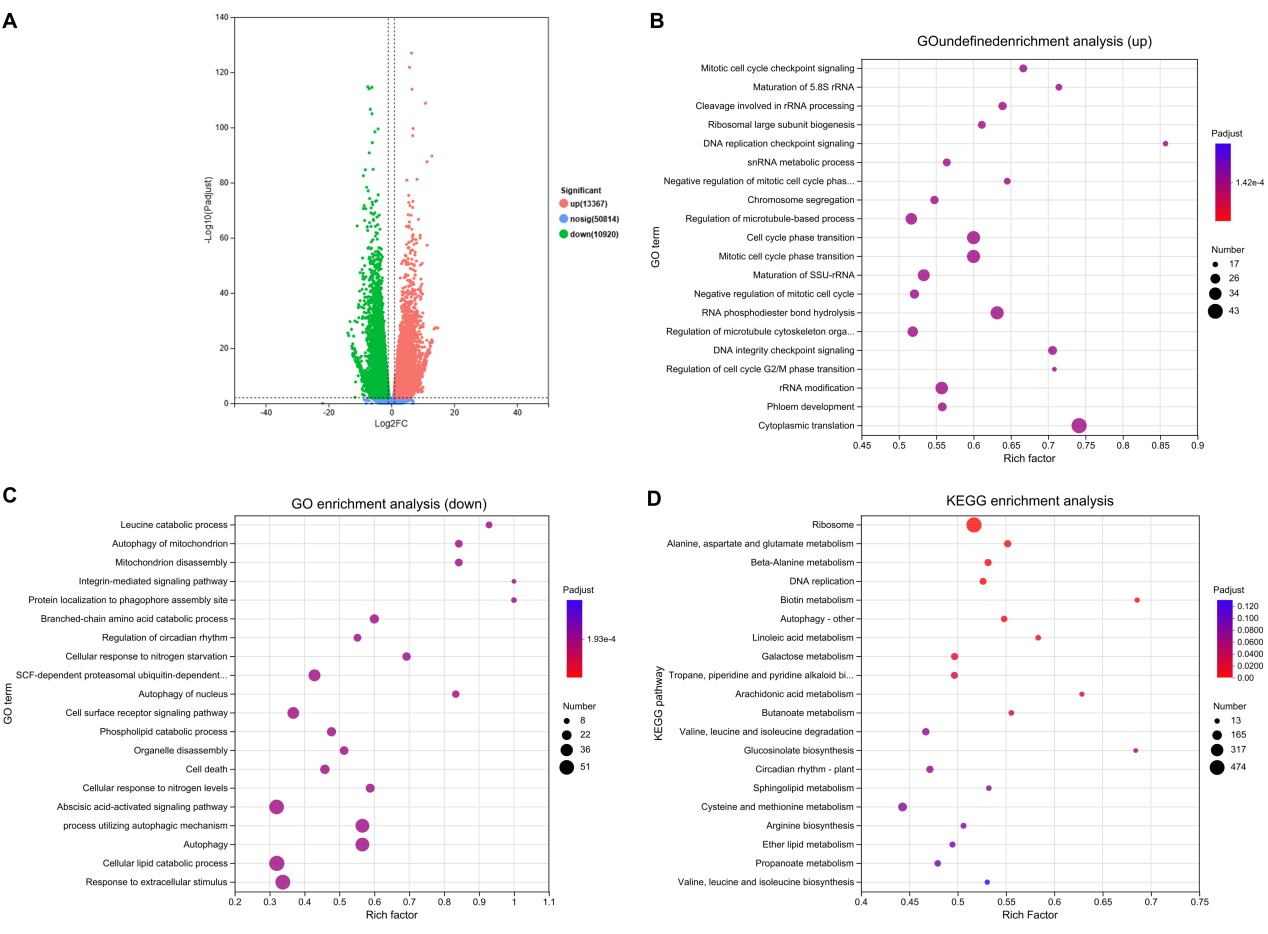


****Fig. S8** Identification of differentially expressed genes (DEGs) between *AhPUGN1.1*-KD and control roots. A** Volcano plot of DEGs between empty vector control and *AhPUGN1.1*-KD roots, red spots indicate significantly up regulated genes, blue spots indicate significantly down regulated genes, and the grey spots indicate genes with non-significant differences. **B,C** GO term enrichment analysis showed the top 20 biological processes enriched in upregulated or downregulated genes. **D** KEGG pathway enrichment analysis of DEGs in *AhPUGN1.1*-KD and control roots.


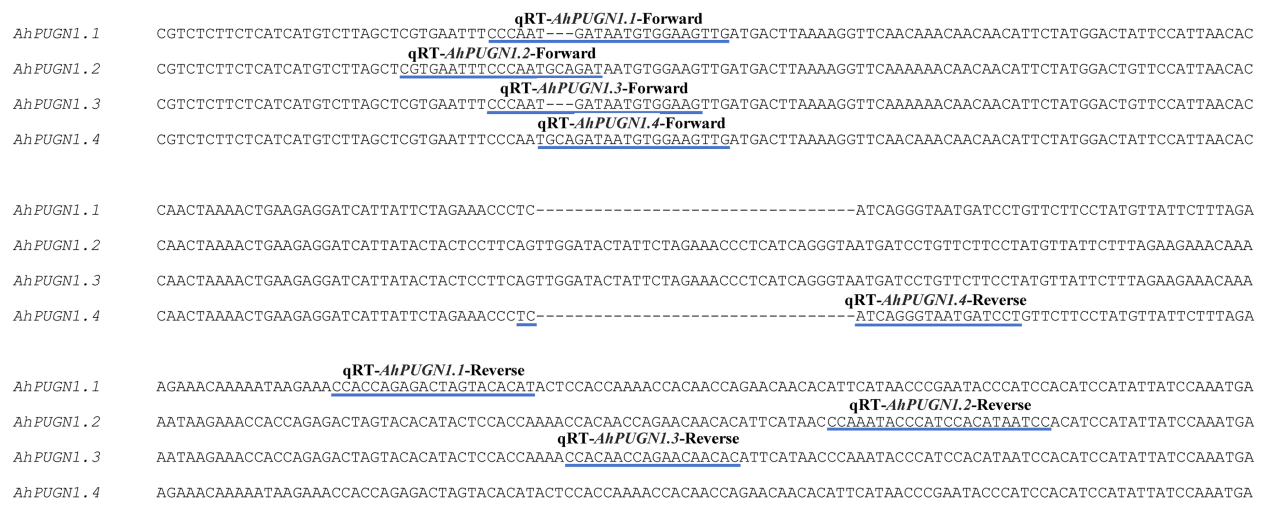


**Fig. S9 qRT-PCR primer positions for *AhPUGN1.1* and its homologous genes.**

**References**

Raul B, Bhattacharjee O, Ghosh A et al (2022) Microscopic and transcriptomic analyses of dalbergoid legume peanut reveal a divergent evolution leading to nod-factor-dependent epidermal crack-entry and terminal bacteroid differentiation. Mol Plant Microbe Interac 35:131-145. https://doi.org/10.1094/MPMI-05-21-0122-R
